# Stroke Care Services in Singapore During COVID-19 Pandemic—A National Perspective

**DOI:** 10.3389/fneur.2020.00780

**Published:** 2020-07-28

**Authors:** Narayanaswamy Venketasubramanian

**Affiliations:** Raffles Neuroscience Centre, Raffles Hospital, Singapore, Singapore

**Keywords:** stroke, services, COVID-19, Singapore, stroke unit

## Abstract

Stroke is a significant cause of admission to Singapore's acute care hospitals. Because of the current COVID-19 pandemic, there have been major changes in the stroke care system. On calling for the public ambulance, those suspected to have COVID-19 infection are taken to the National Center for Infectious Diseases. Otherwise, on arrival at the emergency room, all cases with fever or respiratory symptoms [COVID-19 suspect patients (CSPs)] are evaluated separately by staff wearing full personal protective equipment (PPE). Triage is not delayed. CSPs needing hyperacute therapies are sent to a specially prepared scanner; if not, imaging is deferred to the latter part of the day. CSPs are managed in isolation rooms, and sent to the acute stroke unit (ASU) if two consecutive COVID-19 swabs are negative. Investigation and rehabilitation are done within the room. ASU rounds are attended by essential members, communication by electronic means. Multidisciplinary team rounds have largely ceased, and discussions are via electronic platforms. Patient transfer and staff movement are minimized. All hospital staff wear face-masks, infection control is strictly enforced. Visitors are not allowed; staff make daily calls to update families. Mild stroke patients may be sent home with rehabilitation advice. Out-patient rehabilitation centers are closed. Patients return for out-patient visits only if needed; medications are sent to their home, and nurses make essential home visits. Stroke support and rehabilitation activities have started on-line. Continuing medical education activities are mainly by webinars. Stroke research has been severely hampered. Overall, evidence-based stroke care is delivered in a re-organized manner, with a clear eye on infection control.

## Introduction

The current COVID-19 pandemic has had a significant impact on global economic, political, social, emotional, and medical health. Stroke is a major cause of death and disability throughout the world (1), especially in Asia (2). Stroke occurs in 5.9% of COVID-19 patients, largely ischemic, but with a few hemorrhagic strokes (3, 4). Among patients with COVID-19, cerebrovascular disease is associated with increased mortality and severe COVID-19 infection (5); patients with prior stroke have a more severe COVID-19 infection (6, 7). While there is current interest in hypercoagulable states, vasculitis, and cardioembolism from cardiomyopathy as the mechanism for the stroke, others including large artery atherosclerosis, small artery disease, and other cardioembolic sources such as atrial fibrillation should not be forgotten (8, 9). There has been a noticeable drop in the number of stroke patients arriving at Emergency Rooms (10–12), or they come late (12, 13), or when they are more severe (14). This could be possibly out of fear of entering an environment where there may be COVID-19 patients (15), or due to the reduced availability of ambulances or prompt medical services due to resource diversion to managing COVID-19. All these impact on the provision of evidence-based stroke care that have been proven to reduce death, disability, and stroke recurrence (16).

Singapore is a small tropical island city-state of 5.7 million people situated in the heart of South-East Asia. Stroke is a major cause of death and disability, with an incidence of 1.8/1,000, prevalence of 3.65% among those aged above 50 years, and is among the top 10 causes of hospitalization to our acute care government-funded restructured hospitals that provide heavily subsidized care for more than 95% of acute stroke patients (17).

The number of people diagnosed with COVID-19 in Singapore has been rising (18). The Singapore government quickly established a high-level Multi-Ministry Taskforce on 22 January 2020. It comprises 10 members, all ministers, and is co-chaired by the Minister of Health and the Minister of National Development, with the Deputy Prime Minister as its Advisor. The Taskforce's roles are to direct the national whole-of-government response to the novel coronavirus outbreak; coordinate the community response to protect Singaporeans and stay vigilant against the spread of the disease; and work with the international community to respond to the outbreak. This has resulted in a seamless collaborative response, tapping on the resources of many ministries, so as to swiftly and effectively respond to the infection.

Prior to the COVID-19 pandemic, patients calling for the national ambulance service who were assessed as possibly having a stroke were transported directly to one of three thrombolysis/thrombectomy centers if they met the time windows, or, if not, to the nearest of the seven restructured hospitals or a collaborating private hospital scattered throughout the country. The few patients who arrived via their own transportation means and who met the time windows would receive thrombolysis at whichever center they arrived at, but if they needed thrombectomy, they were then transported to one of the three thrombectomy centers; a small number of thrombectomies were performed at a few private hospitals. Intravenous thrombolysis and thrombectomy services were available 24 h a day. In all hospitals, after emergent triage, patients were, where possible, neuro-imaged while still in the emergency department, before being sent to the acute stroke unit (ASU) of that hospital to be managed by a multidisciplinary stroke team. Those requiring in-patient rehabilitation were transferred to the rehabilitation department or to a nearby community hospital. On discharge, they were followed up by the specialist if necessary, or by the primary care physician. Community-based resources were available including out-patient rehabilitation, home medical and nursing and rehabilitation (19). Stroke support was provided by the Singapore National Stroke Association (SNSA), the oldest national-level support group for stroke survivors and their carers (20, 21).

There have been a few publications with details on how the stroke care has been reorganized due to the COVD-19 situation. Some are hospital-based (22, 23) some only address a specific issue e.g., thrombectomy in that hospital (24). There are no publications on stroke care re-organization at a regional or national level, to my knowledge. In Singapore's response to the COVID-19 epidemic, there were significant changes to the well-coordinated stroke care system, bolstered by lessons learnt from the 2002–2003 SARS-CoV-1 epidemic. This paper aims to present these changes, and what efforts have been made to maintain the provision of high-quality care for those with acute stroke and after discharge, as well as ancillary stroke activities at a national level. The information may be valuable to clinicians, administrators and policy makers involved in stroke care coordination beyond a single hospital, involving hospital and care networks.

## Pre-Hospital

At a national level, on calling for the public ambulance, patients are screened for possible COVID-19 infection or if they are at high risk of having COVID-19 (HrCP). The definition includes return from a country with high numbers of COVID-19 patients, in close contact with persons who have COVID-19 (e.g., same household as, cared for, or exposed for more than 30 min within two meters of a COVID-19 patient), or have been served a quarantine, leave of absence or stay-home order due to contact with a COVID-19 patient. These HrCP are taken directly to the National Center for Infectious Diseases (NCID) where they are assessed and isolated for further care. This policy of sending HrCP to the NCID may be reviewed if the center gets overloaded with patients. There have been no noticeable delays in emergency service response times—the public ambulance service is centrally coordinated and has adequate staff and necessary ambulances; the provision for dealing with large numbers of ambulance requests has been in place for many years, probably based on prior experiences with large disasters and tragedies causing mass casualties. All others who meet the criteria for intravenous thrombolysis or thrombectomy are still taken to the three dedicated centers; if they do not meet the criteria, they are taken to any of the restructured hospitals, as before. Ambulance staff wear full PPE at all times. Each ambulance is fully equipped with adequate stores so that this can be achieved. The staff are all trained on how to quickly don their PPE with minimal delay. Patients may still choose to use their own transportation means for getting to their preferred hospital. There is no practice change here.

## Emergency Room, Neuroimaging and Hyperacute Therapies (Figure 1)

At a hospital level, existing stroke pathways had to be modified in each hospital to meet the needs for strict infection control. In all hospitals, on arrival at the emergency room, all cases are screened again for possible COVID-19 infection and fever; those who are HrCPs are immediately sent to NCID—again this policy may be amended. The NCID is located next to the National Neuroscience Institute (NNI); the neurologist is able to see the stroke patient immediately. NCID has a designated CT scan negative-pressure room that is staffed 24 h a day and used only for COVID-19 patients. By protocol, all thrombectomy patients are prophylactically intubated and sent to a pre-specified operating theater which has negative pressure and a separate ventilation system. They are subsequently managed in the NCID Intensive Care Unit, and later in the NCID wards. The NNI neurologist consults on the patient on a daily basis to collaborate on stroke patient care. It is unlikely that acute stroke therapy was delayed by pre-triage to or subsequent management in NCID (25).

**Figure 1:**
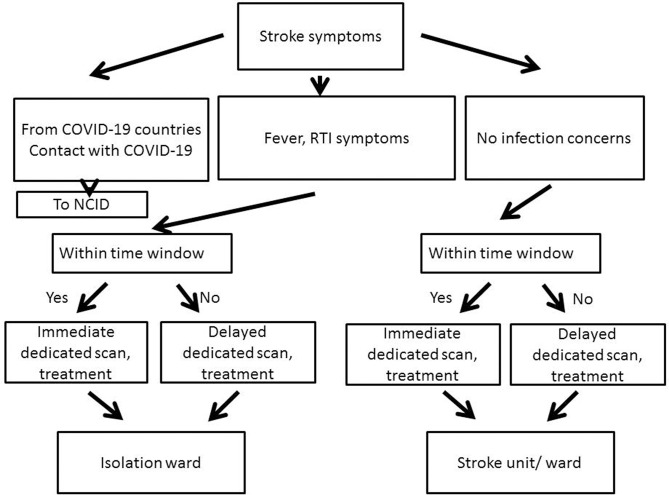
Workflow in the Emergency Department. All patients arriving within the time window for hyperacute therapy are sent for immediate imaging; those who arrive beyond the time window are scanned later at the next available slot, usually later that same day. This applies to both with and without infection concerns (NCID, National Center for Infectious Diseases; RTI, Respiratory Tract Infection).

Those with fever or respiratory tract infection symptoms (e.g., cough, breathlessness, sore throat, runny nose) are defined as COVID-19 suspect patients (CSPs) and are evaluated separately in an area set aside for this purpose, with staff wearing PPE, and the patient in a face mask (FM). Triage, assessment for urgent therapies, imaging are performed without delay for all CSPs and non-CSPs as per the hospital stroke protocol. CSPs needing hyperacute therapies are sent to a scan machine in a minimally equipped room prepared for them, and the scanning room is thoroughly disinfected after each patient; if not for hyperacute therapy, the imaging is delayed to the latter part of the day. The three hospitals with the dedicated thrombolysis-thrombectomy services are able to reserve a scan room just for CSPs. Telemedicine is used where possible to reduce staff members entering this area.

Intravenous thrombolysis commences in the scan room, if possible, for all patients. CSPs who need thrombectomy are prepared in a negative-pressure room if available, and aerosol-generating activities are minimized. Non-CSPs are managed in the usual manner. There is a pool of interventional radiologists and trained staff, allowing for multiple thrombectomy teams in each of the three dedicated centers, in order to cater for the eventuality that if one EVT team unfortunately encounters a confirmed COVID-19 case without adequate protection, that whole team may need to be quarantined for many days.

## Acute Stroke Unit/Isolation Room

CSPs in all hospitals are managed in isolation rooms, usually within a ‘fever ward' or if possible within the acute stroke unit (ASU), with staff in PPE, patients in FM. CSPs are swabbed for COVID-19 daily, and only sent out to the ASU for further care if two consecutive swabs are negative—the results of each swab are ready within 24 h; if positive, the patient is transferred to the NCID. Venepunctures, x-rays, neurosonology, echocardiography, arrhythmia monitoring, and rehabilitation are done within the isolation room by dedicated technicians and staff, based on the clinical need, but may be deferred until the patient has been moved to the ASU if it's less urgent. If repeat imaging is needed, the CSP is transported wearing a FM to the specially-prepared scanner, with imaging performed in the latter part of the day whenever possible. Non-CSPs are managed as usual in the ASU, and have their investigations performed in the usual venues e.g., radiology department, neurosonology laboratory, cardiac laboratory (Table 1).

**Table 1:** Care in isolation room vs. acute stroke unit.

	**Isolation room**	**Acute stroke unit**
Patient	Face mask	Face mask +/–
Staff	PPE when entering patient's roomFace mask when not in patient's room	Face mask
Blood tests Electrocardiography Telemetry set-up	Bedside	Bedside
Neurosonology	Bedside	Neurosonology laboratory Radiology department
Echocardiography, Holter set-up	Bedside	Cardiac laboratory
Chest x-ray	Bedside	Radiology department
Neuroimaging	Scan performed in dedicated scanner later in the day	Scan performed as schedule allows
Rehabilitation	Bedside	Bedside Rehabilitation center

ASU daily rounds are attended only by essential members, communication is by electronic means wherever possible, as all hospitals have electronic medical records including review of imaging and laboratory test results. Multidisciplinary team rounds have largely ceased; discussions are held via electronic platforms. Patient transfer and staff movement are minimized, with ward-based teams where possible. All hospital staff wear FM, infection control is strictly enforced especially hand-washing; social distancing enforced as far as is practicable. Care pathways continue to be followed. Non-urgent surgeries have been postponed.

Visitors are not allowed, except perhaps if the patient is in intensive care (one named visitor throughout hospital stay), or if caregiver training is being provided pre-discharge. Doctors and nurses call the patient's family daily with updates, patients are allowed easy access to ward telephones, wi-fi is provided free where possible. Hospital visits by volunteer befrienders from the SNSA have been halted, but communication over the phone may continue.

Rehabilitation is provided as before, but with social distancing, with patients kept at least one meter apart in the gyms. Transfers to rehabilitation units and community hospitals may be delayed by repeat screening for COVID-19 infection; mild strokes may be sent home with rehabilitation advice.

## Post-Discharge Care

Out-patient rehabilitation centers are closed, which may increase functional limitations and hinder recovery (26). Online rehabilitation services are being tried, but elderly patients are usually unable to manage the required steps, further challenged by their physical disabilities. Traditional Chinese Medicine services have ceased. At the patient level, patients return for out-patient visits only if needed; many are fearful. They may still visit their family physicians. Home visits by nurses are performed where necessary (e.g., to change nasogastric tubes, urine catheters and dressings). Doctors call selected patients to determine progress. Medications are sent to the patient's home for a small fee to maintain compliance. Teleconsultation is available but not actively taken up by elderly patients. Some stroke support activities by SNSA have started on-line e.g., exercises, aphasia therapy, but again disabled elderly who are not familiar with the use of online services may not participate.

## Professional Matters

All healthcare professionals are regularly recertified by their respective professional boards (e.g., Singapore Medical Council for doctors), usually by participating in continuing medical education (CME) activities (27). Professional recertification requirements have not been relaxed—full-practice doctors still need to earn at least 50 points over 2 years. But the availability of CME activities by electronic means via webinars has greatly increased; COVID-19 CMEs are popular and well-attended. Stroke research has been severely hampered as subjects are fearful to come to hospital, movements around the hospital is strictly controlled. But some researchers are taking the opportunity provided by reduced out-patient work to write their previously-shelved papers.

## Other Solutions

There have been a number of publications of stroke systems of care during the COVID-19 epidemic. Pre-hospital triage, advance notice by the ambulance to the Emergency Room, adequate training and use of PPE to reduce staff infection, adequate respiratory management en-route, care in appropriately equipped hospitals, and minimizing transfers is important (28, 29). Existing stroke pathways may need to be revised (23), including for endovascular therapy (30, 31). Rehabilitation should not be neglected (32, 33). It can be managed with stream-lined protocols, use of telemedicine/telerehabilitation, attending to COVID-19-related adverse events (such as fever and respiratory symptoms), enforcing social distancing and adequate sterilization of equipment (34, 35). While trying to provide the best of care to patients, staff safety cannot be neglected (36). A protocol specifically for the management of stroke among patients with COVID-19 may be helpful (22) and needs to be practiced (37). In effect, the entire system of care may needs to be reorganized (38). Guidelines and suggestions for stroke care have been proposed (39–44), but each center had best develop its own or tailor existing guidelines to meet and fit its needs. Consent for research, usually performed face-to-face, may be taken remotely (45), either electronically or by phone; follow-ups may need to be by phone (46). Challenges for stroke care are even greater in developing countries (47).

## Conclusions

The COVID-19 pandemic has posed some challenges to the provision of stroke care in Singapore. There is no overall change in pre-hospital and hyperacute stroke care policies, but CSPs are cared for in isolation; stroke support services and stroke research are majorly affected. Still, evidence-based stroke care is delivered in a re-organized manner, with a clear eye on infection control. The future is likely to see the greater use of electronic communication and telemedicine.

## Author contribution

NV wrote the paper and agrees to be accountable for the content of the work.

## Conflict of Interest

The author declares that the research was conducted in the absence of any commercial or financial relationships that could be construed as a potential conflict of interest.
